# LIFESPAN CANNABIS USE PATTERNS IN OLDER USERS

**DOI:** 10.1093/geroni/igz038.737

**Published:** 2019-11-08

**Authors:** Sara H Qualls, Kanika arora, Julie Bobitt, Brian Kaskie

**Affiliations:** 1 University of Colorado Colorado Springs, Colorado Springs, Colorado, United States; 2 University of Iowa, Iowa City, Iowa, United States; 3 University of Illlinois at Urbana-Champaign, Champaign, Illinois, United States

## Abstract

The rapidly rising rates of cannabis use among older adults may reflect a rise in late-onset users, re-engagement after a period without use, or a continuous use pattern since young adulthood that is more visible after legalization of cannabis. Older (age 60+) cannabis users (n=82) provided retrospective ratings on their frequency of use across adulthood. Approximately 28% were not using cannabis when young adults, with a larger percentage (40%) reporting non-use while ages 31-49 and 37% reported non-use when ages 50-64. Approximately 21% of older users were first time users, with 60% low frequency and 35% daily/weekly users. High frequency users generally were high frequency users throughout adulthood, but the pattern varied substantially by gender and mode of consumption. Women were more likely first-time users than men, and more likely non-smokers. Among non-smokers, about 40% were first-time users. Implications are explored for research, policy, and clinical practice.

